# Transcriptomic and computational analysis identified LPA metabolism, KLHL14 and KCNE3 as novel regulators of Epithelial-Mesenchymal Transition

**DOI:** 10.1038/s41598-020-61017-y

**Published:** 2020-03-06

**Authors:** V. Di Lollo, A. Canciello, M. Orsini, N. Bernabò, M. Ancora, M. Di Federico, V. Curini, M. Mattioli, V. Russo, A. Mauro, C. Cammà, B. Barboni

**Affiliations:** 1 Faculty of Bioscience and Technology for Food, Agriculture and Environment, University of Teramo, Teramo, Italy; 2 Molecular biology and genomic Unit, Istituto Zooprofilattico Sperimentale dell’Abruzzo e del Molise “G. Caporale”, Teramo, Italy

**Keywords:** Stem-cell biotechnology, Mechanisms of disease, High-throughput screening, Transcriptomics

## Abstract

Epithelial-mesenchymal transition (EMT) is a complex biological program between physiology and pathology. Here, amniotic epithelial cells (AEC) were used as *in vitro* model of transiently inducible EMT in order to evaluate the transcriptional insights underlying this process. Therefore, RNA-seq was used to identify the differentially expressed genes and enrichment analyses were carried out to assess the intracellular pathways involved. As a result, molecules exclusively expressed in AEC that experienced EMT (GSTA1-1 and GSTM3) or when this process is inhibited (KLHL14 and KCNE3) were identified. Lastly, the network theory was used to obtain a computational model able to recognize putative controller genes involved in the induction and in the prevention of EMT. The results suggested an opposite role of lysophosphatidic acid (LPA) synthesis and degradation enzymes in the regulation of EMT process. In conclusion, these molecules may represent novel EMT regulators and also targets for developing new therapeutic strategies.

## Introduction

Epithelial-mesenchymal transition (EMT) is a complex biological process. Although intracellular pathways leading to the trans-differentiation of epithelial into fibroblastic-like cells are known, several molecular mechanisms still remain to be clarified despite the clinical urgency of the issue^1^. At the present, EMT is recognized to play an essential role in driving three different biological events: (1) type 1 EMT during embryo development, (2) type 2 in the fibrotic process, wound healing and tissue regeneration while (3) type 3 during metastatic process^2^. Regardless of the type, EMT is triggered by initiator molecules (e.g. cytokines and growth factors) able to induce the so-called EMT master regulators (Snail, Twist and Zeb, among the others) with the consequent loss of epithelial markers (primarily E-Cadherin) and the acquisition of mesenchymal ones^3^. During this transition, a number of transcriptional changes occur into epithelial cells. Among these, the expression of mesenchymal genes leads to the rearrangement of cytoskeletal components, increased migration, secretion of enzymes responsible of the extracellular matrix degradation, changes in the lipid bilayer fluidity and shifts in metabolic pathways^4^.

Emerging evidences demonstrated that some stem cells and cancer cells share a number of biological processes and amongst them the ability to undergo EMT^5^. Indeed, it has been reported that epithelial stem cells that undergo EMT also acquire cancer stem cell-like features like invasiveness, high migratory phenotype and cell-contact independent growth^3,5^. Similarly, primary neoplastic cells that undergo EMT develop stem cell-like characteristics such as self-renewal, cell plasticity and stem cell marker expression^1^. Amniotic Epithelial Cells (AEC), a subset of placental stem cells, can be addressed to experience EMT for redirecting their biological fate^6–9^. Indeed, AEC have been proved capable to interact with different typologies of host damaged tissue such as lung^10^, liver^11^, cholangiocyte^12^, CNS^13^, tendon^14^ or sinusoidal endothelial cells^11^ by promoting regeneration, managing inflammation and replacing damaged cells with functional/mature ones (without cell fusion). The level of maturation implies either a tissue replacement as epithelial cells^15–21^, or as mesenchymal cells by experiencing EMT^9,14,22–24^. This latter EMT plasticity of AEC has been demonstrated *in vitro* and *in vivo* more extensively in animal (isolated at earlier stage of gestation)^9^ that human models^25^.

During AEC in *vitro* expansion, EMT is avoided by adopting specific cultural protocols^11,26^ in order to preserve their native key functional attitude such as stemness, plasticity and immunomodulatory activity^26,27^. In particular, the mesenchymal transition of AEC can be controlled by progesterone (P_4_) that exerted a powerful inhibitor role by interfering with the TGF-β1 signaling pathways. When cultured in the presence of P_4_, AEC were able to express their self-renewal ability by preserving the native epithelial phenotype that spontaneously would be lost during the *in vitro* expansion^26^. Of note, P_4_-mediated model of EMT inhibition in AEC negatively affects TGF-β1 signaling pathway and also induces the reversion of mesenchymal phenotype similarly to what happens in other EMT models treated in literature^28–30^. Therefore, AEC represent a comparable cell model to study the complex process of EMT.

On the contrary, the phenotype shift has been associated with a favorable step-wise *in situ* differentiation process useful to exploit the therapeutic potential of AEC in mesenchymal tissues verified under both clinical or preclinical settings^26,31,32^. Therefore, revealing the underlying molecular insights of EMT in AEC becomes crucial in order to improve their use in tissue engineering protocols as well as to deepen our understanding of the intracellular pathways of this widespread biological process.

Here the AEC *in vitro* model was used in order to investigate the molecular events underlying EMT. To this aim, RNA sequencing (RNA-seq) has been used to compare the transcriptome between AEC that spontaneously underwent EMT (mesenchymal AEC: mAEC) *vs* cells that maintained their native epithelial phenotype (epithelial AEC: eAEC). The results highlighted discrete transcriptional landscape divergences between the two cell populations that, interpreted with the gene network computational model approach, pointed to new mechanistic insights for the comprehension of EMT and provide novel potential markers for therapeutical strategies in regenerative medicine and oncology.

## Results

### Progesterone prevents AEC *in vitro* epithelial-mesenchymal transition

**Figure 1:**
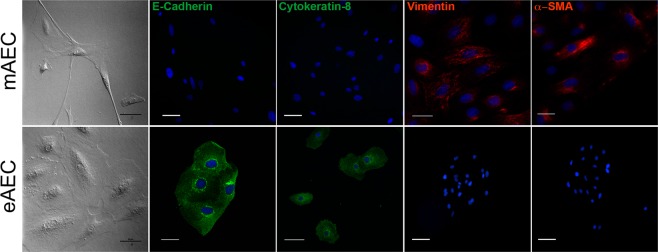
mAEC and eAEC phenotype examples after three passage of *in vitro* amplification. Upper box. AEC cultured using validated amplification protocol (mAEC) showed a fibroblastic-like, elongated morphology, high positivity for mesenchymal markers such as Vimentin and α-SMA and a low expression of epithelial markers. Scale Bar: 50 µm. Bottom box. eAEC cells preserved the native epithelial phenotype and the high expression of epithelial markers. Scale Bar: 50 µm. Conversely, Vimentin and α-SMA showed a rare or absent expression. Scale Bar: 25 µm.

As a consequence of *in vitro* expansion, native AEC underwent EMT by changing their morphology (Fig. 1). Barely in three cultural passages, AEC spontaneously lost their cobblestone shape to acquire an elongated fibroblast-like shape (mAEC). EMT was confirmed by the dramatic loss of epithelial markers (12.5 ± 1.6% of E-Cadherin and 9.8 ± 1.7% of Cytokeratin-8 positive cells) and the acquisition of mesenchymal ones (84.9 ± 2.9% of Vimentin and 87.5 ± 2.4% α-SMA positive cells). Conversely, when AEC were exposed to P_4_, the native epithelial phenotype (eAEC; Fig. 1) was preserved as confirmed by their morphology, the low expression of Vimentin (6.6 ± 1.7% of positive cells) and α-SMA expression (13.7 ± 2.8% of positive cells) and the widespread positivity for E-Cadherin (85.5 ± 1.9% of positive cells) and Cytokeratin-8 (87.3 ± 2.2% of positive cells: Fig. 1). Therefore, three independent cellular replicates of AEC expansion with or without P_4_ was adopted to obtain the two cell populations (eAEC and mAEC) whole transcriptome.

### *In vitro* expanded AEC transcriptional landscape

**Figure 2:**
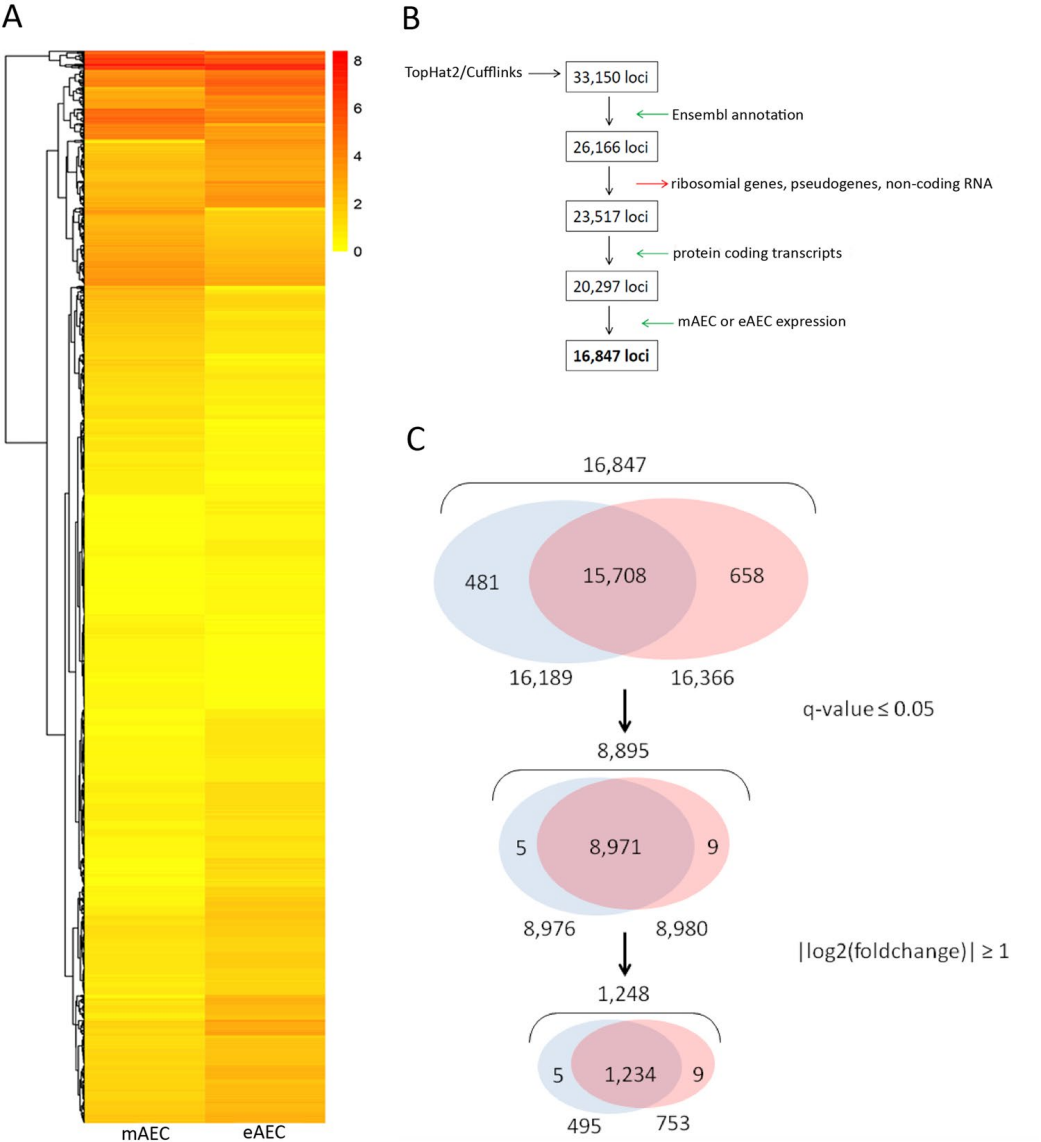
Bioinformatics steps. (**A**) Heatmap analysis shows differences in gene expression between the mAEC and eAEC. Each column represents a cell population and each row represents a gene. The expression levels, based on FPKM expression values, are visualized using a gradient color scheme. **(B)** The flowchart summarizes the procedure performed to identify the study dataset (16,847 *loci*) using TopHat2/Cufflinks pipeline (detailed information on filtering procedure can be found in Supplementary File S1). The boxes represent the subsequent output data returned from individual filtering steps (green and red arrows) starting from RNA-seq raw data (33,150 *loci*). **(C)** The Venn diagrams show the characteristic DEGs number identified in both AEC populations after q-value ≤ 0.05 and |log2(foldchange)| ≥ 1 filtering steps. The figure not only displays the number of overlapped genes between the two cell populations but highlights the genes expressed exclusively in one of them.

RNA-seq analysis was performed on the three mAEC and eAEC replicates resulting in identifying 33,150 expressed *loci* (Fig. 2A). In order to guarantee a high-quality data set, a filtering procedure was adopted as described in Fig. 2B and in Supplementary File S1. This depicted the transcriptional landscapes of mAEC and eAEC, detailing both common (15,708) and populations-specific genes (481 genes in mAEC and 658 in eAEC). Retaining only those genes with confidence (q-value ≤ 0.05) and exhibiting a fold change higher than |log2fold| ≥ 1^33^ (Fig. 2C), a reliable subset of 1,248 differentially expressed genes (DEGs) was identified. In particular, a total of 495 and 753 DEGs were over-expressed in mAEC and eAEC, respectively (Supplementary Dataset S2). Interestingly, 5 and 9 DEGs belonged exclusively to mAEC and eAEC, respectively. In detail, mAEC specifically expressed Sarcolipin (SLN), Trefoil factor 3 (TFF3), Small muscle protein X-linked (SMPX), ENSOARG00000000376 and Chromosome 5 open reading frame 58 (C5orf58) whereas eAEC expressed Kelch like family member 14 (KLHL14), ENSOARG00000003845, potassium voltage-gated channel subfamily E regulatory subunit 3 (KNCE3), Natural killer cells antigen CD94-like, Collagen type IX alpha 1 chain (COL9A1), ENSOARG00000019504, ENSOARG00000010151, ENSOARG00000005173 and ENSOARG00000014113.

### Enrichment analysis

**Figure 3:**
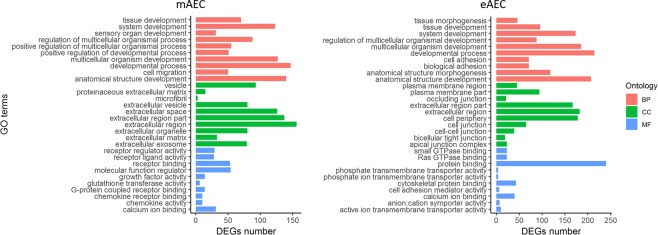
GO enrichment analysis. Representative scheme of the top 10 most abundant GO terms identified for the mAEC and eAEC in the three GO category: Biological Process (red), Cellular component (green), and Molecular Function (blue). The x-axis indicates the number of genes in a specific category while the y-axis indicates different GO terms.

**Figure 4:**
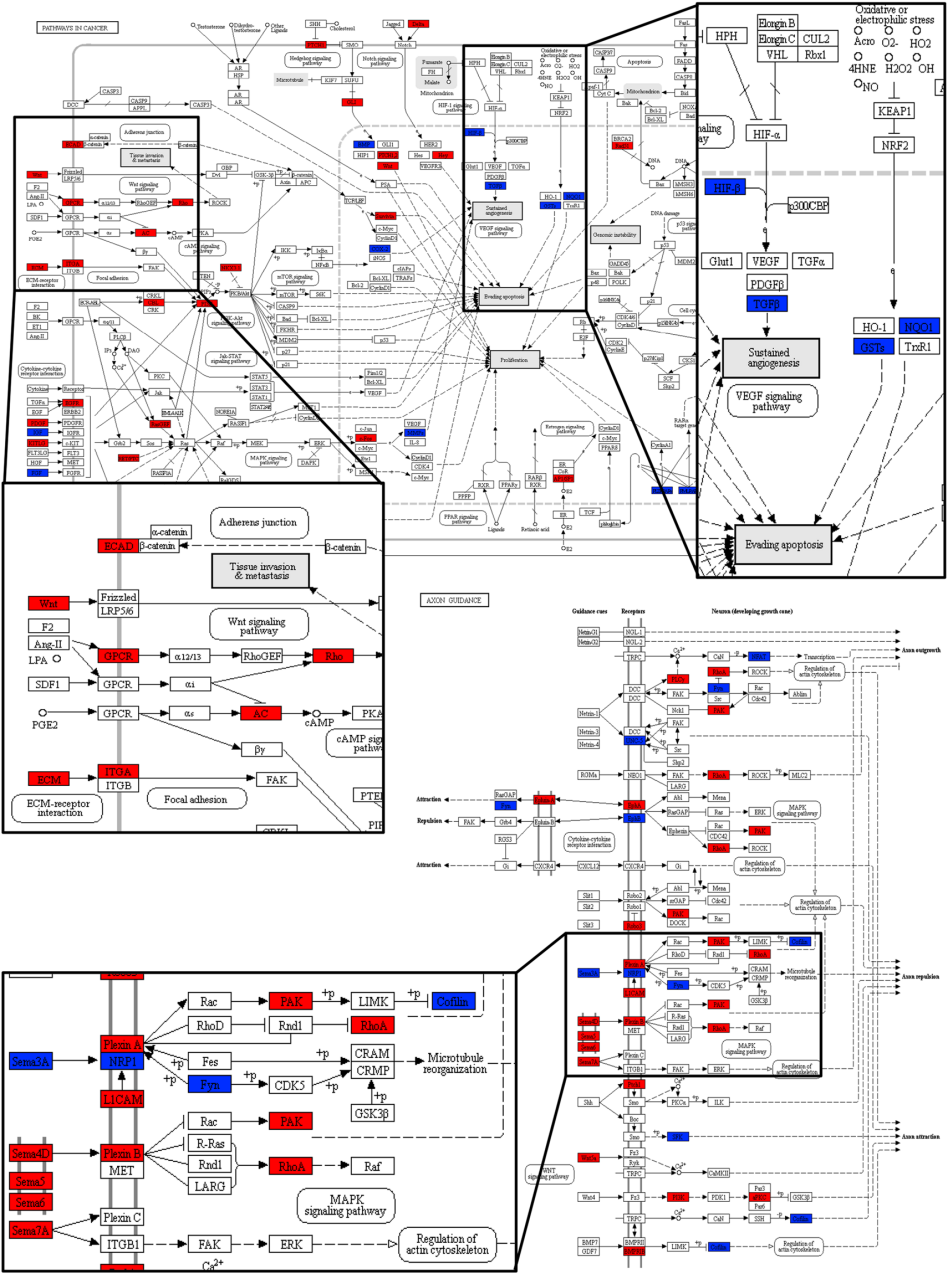
KEGG maps analysis. The figure displays two examples of differentially regulated KEGG Pathways: Pathway in cancer (upper map) and Axon Guidance (lower map). The differentially expressed genes (DEGs) are mapped in blue for mAEC and in red for eAEC cells.

The enrichment analysis was conducted on DEGs for the three GO ontologies (biological process: BP, cellular component: CC, molecular function: MF). As shown in Fig. 3, excluding the elements in common, some differences in GO terms between mAEC and eAEC were identified, highlighting the specificity functional bias of the two cell populations. The BP category shows 5 terms exclusively over-represented in mAEC (4 terms related to regulating development processes and 1 related to cell motility process, Fig. 3) while, in eAEC, terms were mainly associated with anatomical structure development and cell adhesion processes. In CC category, 8 terms referred to extracellular structures (Fig. 3) were enriched in mAEC only, whereas, cell junction-related GO terms marked the eAEC (Fig. 3). In MF category, 9 out 10 terms resulted different between the two populations: terms related to receptor-ligand binding regulation function (n = 8) and transferase enzymatic activity (n = 1) characterized the functional profile of mAEC whereas, in eAEC, 5 terms resulted enriched in protein binding functions and the remaining 4 are related to transmembrane transport activity. Further, functional Kyoto Encyclopedia of Genes and Genomes (KEGG) pathways enrichment analysis was performed. As result, 68 pathways significantly enriched between the two populations were detected: 29 pathways in mAEC and 39 in eAEC. Five pathways resulted commonly enriched in both conditions (Axon Guidance, Pathways in cancer, Fluid shear stress and atherosclerosis, Ras signaling pathways and Protein digestion and absorption). Intriguing, mAEC and eAEC showed a divergent gene expression pattern in these common enriched pathways, thus suggesting an opposite regulation of the same molecular processes (Fig. 4).

### Gene-gene interaction networks for the identification of control mechanisms

**Figure 5:**
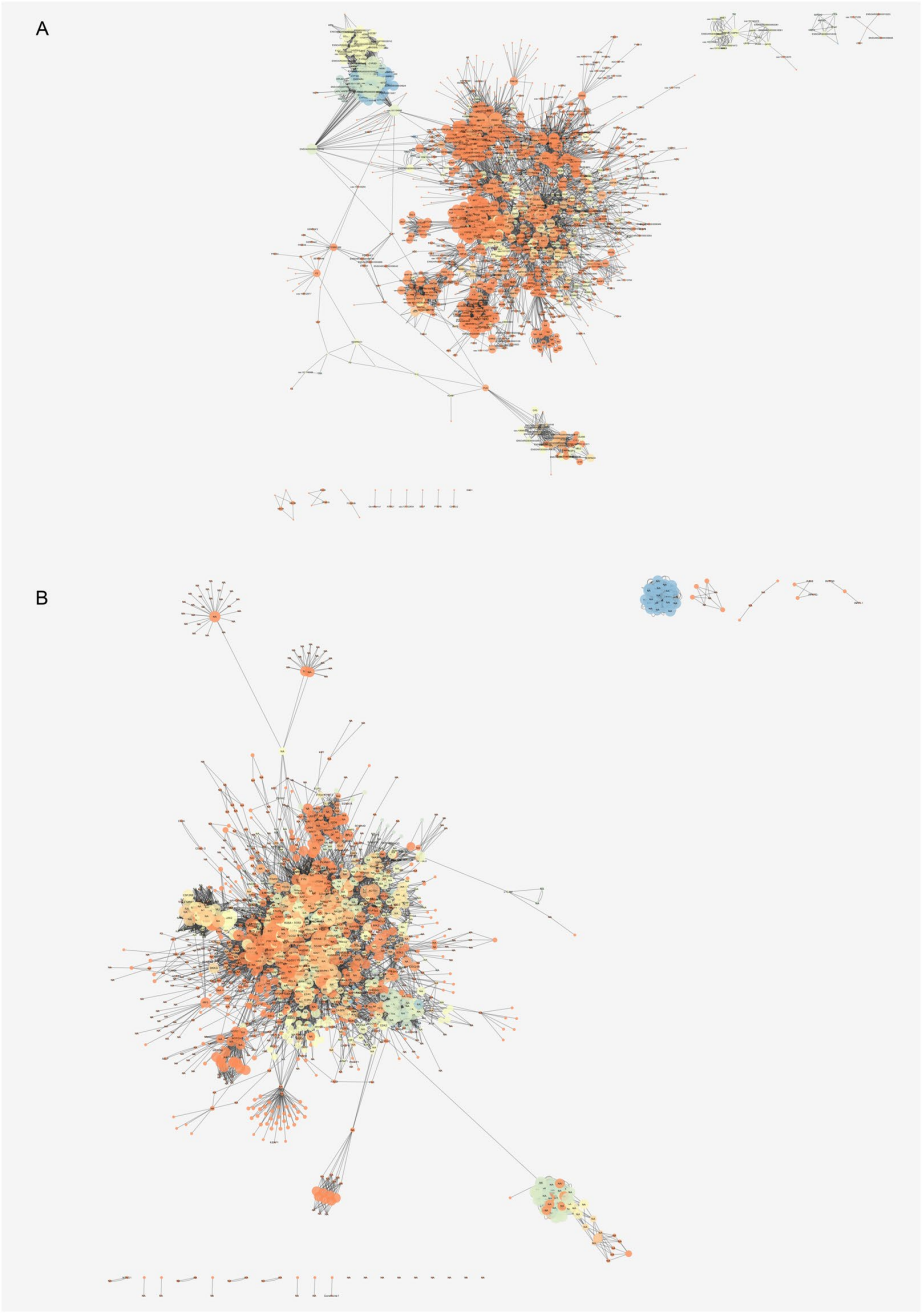
Gene-gene interaction network analysis. The mAEC (**A**) and the eAEC (**B**) networks were displayed using the Cytoscape Prefuse Force Directed Layout. In the figure the size of nodes is directly proportional to the node degree and the gradual color change reflects different clustering coefficient values.

The gene-gene networks were built using nodes and edges obtained from the 29 and 39 KEGG pathways identified in mAEC and eAEC, respectively (Fig. 5A,B). The mAEC network consisted of 1,434 nodes and 10,320 edges while eAEC network comprised 1,807 nodes and 14,290 edges. By focusing on the main connected components (MC) only, as representative of the entire networks^34^, two subnetworks, henceforth named MC_mAEC and MC_eAEC, were extracted clustering 1,375 mAEC nodes (95.9%) and 1,736 eAEC nodes (96.1%), respectively (Table 1). According to Barabási-Albert (BA) model^14,15^ both networks could be classified as scale-free^34,35^. A total of 149 and 203 hubs, defined as those node highly connected^36^, were found in MC_mAEC and in MC_eAEC, respectively (Supplementary Dataset S3).

**Table 1:** Gene-gene interaction network analysis.

		MC_mAEC	MC_eAEC
Parameters	Number of nodes	1375	1736
	Number of edges	10209	13703
	Clustering coefficient	0.070	0.071
	Connected component	1	1
	Network diameter	16	16
	Shorts paths	589307 (31%)	156561 (51%)
	Characteristic path length	6.123	5.362
	Avg. Number of neighbors	13.785	15.202
Node degree distribution (in degree/out degree)	-γ	−1.220/−1.340	−1.378/−1.454
	R	0.861/0.775	0.816/0.838
	R2	0.628/0.663	0.701/0.716
Clustering coefficient *vs* Node degree	-γ	−0.417	−0.466
	R	−0.160	−0.164
	R2	0.035	0.176

The table shows the MC_mAEC and MC_eAEC topological parameters analysis.

From the Kernel Density Estimation (KDE) analysis 23 and 9 hubs in mAEC and eAEC, respectively, displayed the functional characteristic of local hubs (Supplementary Dataset S4). Most of mAEC local hubs resulted being isoforms of the glutathione transferase (GST) superfamily, which groups different classes of genes with a crucial role in cell protection against oxidative damages^37^. Among them, GSTM3 and GSTA1-1 showed a significant up-regulation. Conversely, eight out nine eAEC local hubs (LPINs 1–3 and PLPPs 1–5) resulted all members of the phosphatase/phosphotransferase family and their function is associated with lipid biosynthesis process^38^.

In each cell population network, 200 bottlenecks, defined as those nodes having many “shortest paths” going through them^39^, were selected, according to Ordinelli *et al*.^36^. Analyzing the two bottleneck lists (Supplementary Dataset S5A–B), some of them resulted DEGs, then supporting their topological importance within the AEC networks.

**Figure 6:**
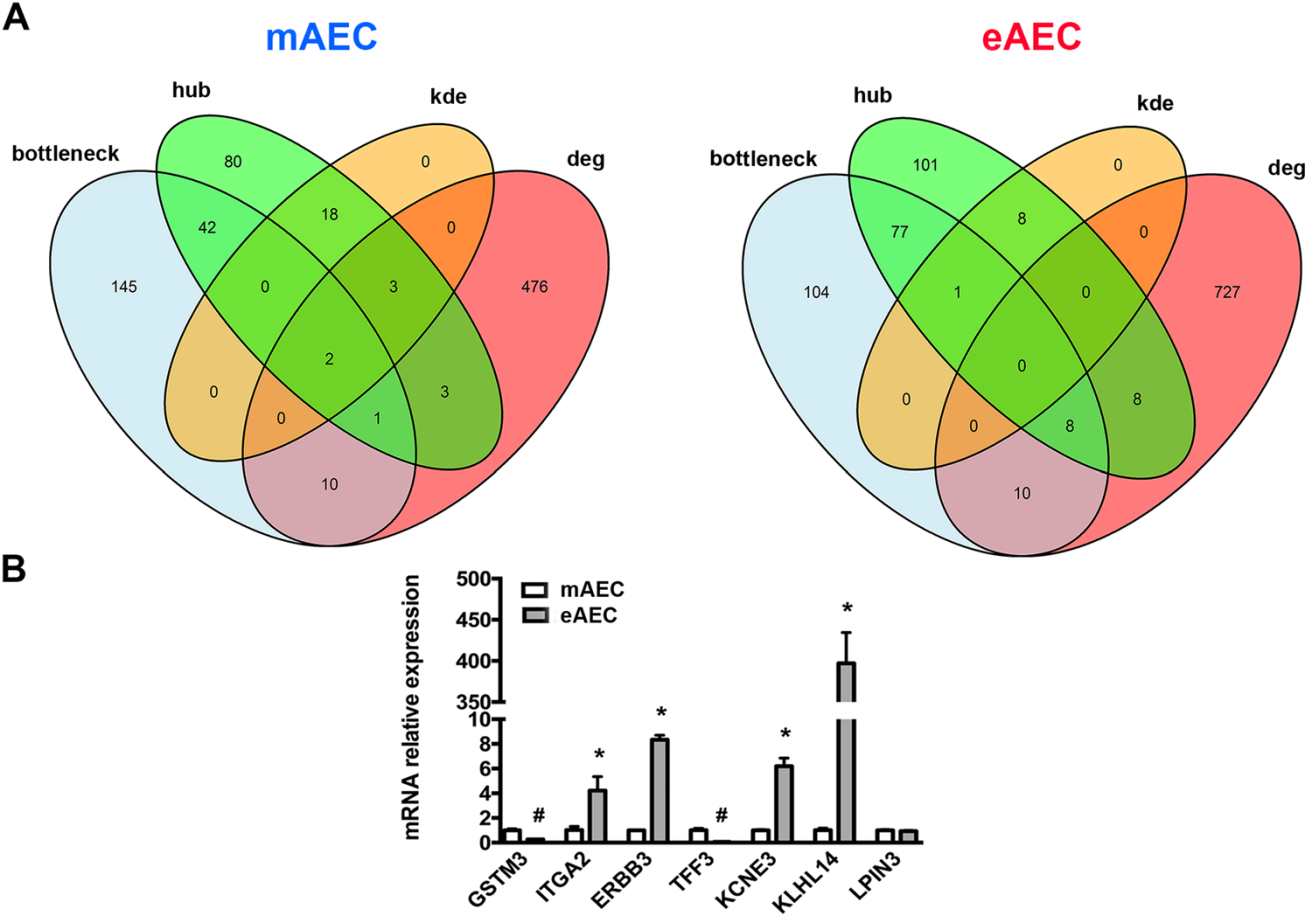
Sub-networks analyses. (**A**) Venn diagram analysis of overlapping genes in mAEC and eAEC. **(B)** Real-Time qPCR validation of most representative genes. Results are the mean ± SEM, from n = 3 independent experiments performed in triplicate. ^#^represents a significative reduction in eAEC, with p < 0.001; *represents a significative increase in eAEC, with p < 0.001.

To better identify genes depicting eAEC and mAEC transcriptional framework, bottlenecks items, cluster density results (KDE) and differential expression analysis were integrated (Fig. 6A). First of all, hubs-bottlenecks genes (45 and 86 in mAEC and eAEC, respectively) were matched with DEG lists, thereby identifying 3 out of 495 DEGs that belonged to the bottleneck-hub subset in mAEC and 8 out of 753 DEGs that were eAEC specific. More in detail, mAEC specific bottleneck-hub subset comprised Microsomal glutathione-S-transferase 1-1, Glutathione S-transferase mu 3 (GSTM3) and Ras protein specific guanine nucleotide releasing factor 1 (RASGRF1). On the other hand, eAEC specific bottleneck-hub subset included Erb-b2 receptor tyrosine kinase 3 (ERBB3), Integrin subunit alpha 2 (ITGA2), Mitogen-activated protein kinase 13 (MAPK13), Phospholipase C gamma 2 (PLCG2), Epidermal growth factor receptor (EGFR), Ephrin A5 (EFNA5), ENSOARG00000000422 and Ras homolog family member A (RHOA). Finally, by intersecting these subsets with the genes emerged from KDE analysis, it was possible to identify the presence of two local hubs differentially expressed within mAEC (Microsomal glutathione-S-transferase 1-1 and Glutathione S-transferase mu 3). Conversely, in eAEC it was identified only one local hub (Lipin 3: LPIN3) showing bottleneck features but not differentially expressed in the two cell populations.

### Real-time qPCR validation of the new identified controller genes

In order to investigate donor to donor variability quantitative Real-Time PCR was performed by using triplicate of amplified AEC derived from 3 different fetuses. Thus, genes to analyze were chosen among those exclusively expressed in a given condition (referred as eAEC or mAEC “specific DEGs”), those presenting simultaneously characteristics of bottleneck, hub, DEG, KDE or a combination of these features. In detail, for mAEC were selected GSTM3 (a gene with bottleneck-hub-DEG-KDE features) and TFF3 (mAEC_specific DEGs) (Fig. 6B). Conversely, for eAEC condition were selected ITGA2, ERBB3 (both genes with bottleneck-hub-DEG features), LPIN3 (a gene with bottleneck-hub-KDE features), KCNE3 and KLHL14 (both eAEC_specific DEGs) (Fig. 6B). qPCR analysis confirmed that GSTM3 and TFF3 were significantly increased in mAEC condition respect to eAEC (Fig. 6B). Analogously, ITGA2, ERBB3, KCNE3 and KLHL14 transcripts were significantly over-expressed in eAEC respect to mAEC whereas LPIN3 showed similar expression levels between these two conditions (Fig. 6B). These results seemed to indicate GSTM3 and TFF3 as key EMT-inducers operating in mAEC whereas ITGA2, ERBB3, LPIN3, KCNE3 and KLHL14 as essential epithelial genes for the inhibition EMT in eAEC.

### Identification candidate genes for the role of EMT driver genes

Among the genes identified by the different analyses, we focused on well-specific group (Table 2). In particular, GSTA1-1, GSTM1 and GSTM3 were selected as local hubs of mAEC networks whereas LPIN1, LPIN2 and PLPP2 as local hubs of eAEC networks, though they only showed a slight upregulation in mRNA expression (|fold change| = 0.320077, 0.233158 and 0.457677, respectively). These latter genes belong to a common metabolic pathway of an important lipid mediator called lysophosphatidic acid (LPA)^40^. More in detail*, LPINs* and *PLPPs* encode for genes involved in the degradation of LPA^38,41^. Nevertheless, LPA acting through its membrane receptor (LPAR) is able to active the nuclear translocation of nuclear factor erythroid 2-related factor 2 (Nrf2)^42^. Intriguingly, GSTs are among the main target genes of Nrf2. Finally, a connection was found between the LPA pathway and the two most up-regulated genes in eAEC population, Kelch-like gene family 14 (KLHL14) and a potassium (K^+^) channel regulatory β subunits (KCNE3). The former (KLHL14) belongs to the same family of molecules that negatively regulate Nrf2 function, whereas the transcriptional regulation of the latter (KCNE3) seems to be Nrf2-mediated^43–46^. Altogether these results provided strong evidence of novel putative metabolic pathway comprehending the identified genes which could be involved in the regulation of EMT process.

**Table 2:** Key driver genes.

		Topological features		Expression features	
Symbol	Functional Annotation	Local hub	Bottleneck	DEG	Significant
**GSTM3**	Plays a role in cell protection against oxidative damages; involved in tumor progression^37,52^	x	x	x	x
**GSTA1**	Takes part in detoxification process; associated with tumor progression^69–71^	x			
**GSTM1**	Important role in Phase II detoxification process^37,72^	x		x	x
**TFF3**	Maintains the integrity of the gastrointestinal tract; promote proliferation, invasion and EMT^73–75^			x	x
**ITGA2**	Regulates cytoskeletal organization and cellular motility; implicated in stem cell differentiation^76,77^		x	x	x
**ERBB3**	Linked to cancer etiology and progression; its role in EMT is still under debate^78,79^		x	x	x
**LPIN1**	Involved in phospholipid and triacylglycerol synthesis; catalyzes the dephosphorylation of phosphatidate to diacylglycerol (DAG)^80,81^	x			
**LPIN2**	Plays central role in lipid metabolism; catalyzes the dephosphorylation of phosphatidate to diacylglycerol (DAG)^80,81^	x			
**LPIN3**	Has a role in lipid metabolism; expressed in the gastrointestinal tract and liver^38,80,81^	x	x		
**PLPP2**	Involved in lysophosphatidic acid degradation^38,41^	x			x
**KCNE3**	Modulates the voltage-gated potassium (Kv) channels gating^56,82^			x	x
**KLHL14**	Participates in extracellular communication/interaction, cell morphology and actin binding regulation^43^			x	x

List of genes involved in the regulation of EMT process in mAEC and eAEC. For each of these genes the main biological functions have been reported. The table also shows the topological and the expression features identified from computational analyses.

## Discussion

Here a novel putative pathway involved in the regulation of EMT process was identified by using AEC as an *in vitro* model. Recently, it has been developed an innovative protocol to control AEC phenotype during *in vitro* amplification, in particular for regenerative medicine purposes^47^. Exploiting this protocol, two population of AEC with different phenotypes were obtained: epithelial (eAEC) and mesenchymal (mAEC) cells. Thereafter, by combining the transcriptomic analysis with the systems biology approach, a discrete number of inter-connected genes have been identified as controllers of EMT process in AEC.

**Figure 7:**
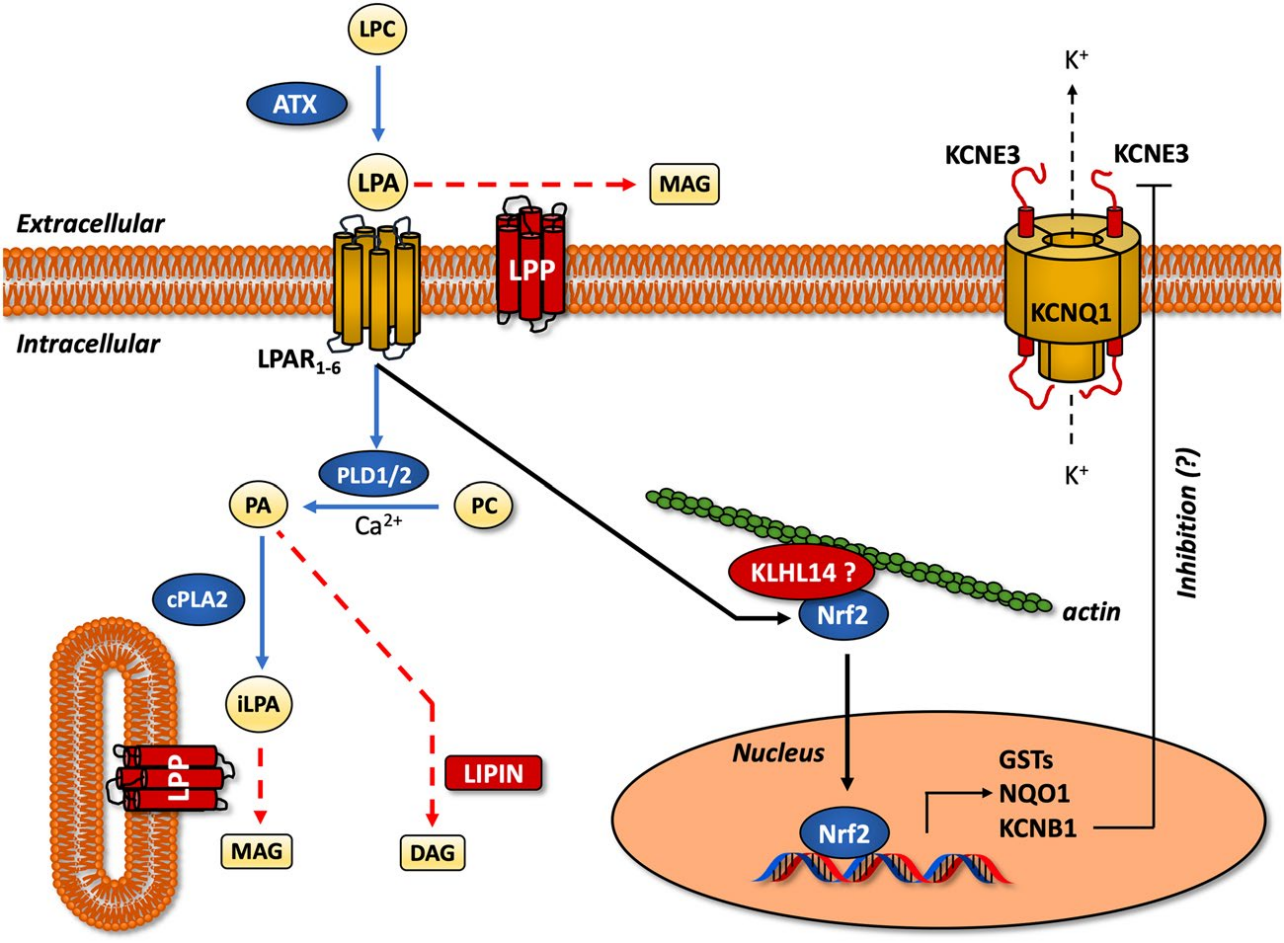
LPA metabolism, KLHL and KCNE3 as novel EMT controller genes. Schematic pathway representation of controller genes involved in the induction (blue) and in the inhibition (red) of EMT in AEC cell model. Moreover, biosynthetic (blue arrows) and degradative (red arrows) LPA pathways are showed. KLHL14 is hypothetically represented in a complex with Nfr2, as its homologous protein KLHL19. LPC, lysophosphatidilcoline; LPA, lysophosphatidic acid; iLPA, intracellular LPA; ATX, autotaxin; LPAR, LPA receptor; LPP, lipid phosphate phosphatases; PDL, phospholipase D; PLA2, phospholipase A2; PC, phosphatidylcholine; PA, phosphatidic acid; MAG, monoacylglycerol; DAG, diacylglycerol; KLHL14, kelch-like protein 14; Nrf2, nuclear factor erythroid 2-related factor 2; GST, Glutathione S-transferase; NQO1, NAD(P)H:quinone oxidoreductase. See the text for more details.

Transcriptomic data strongly suggest a role of lysophosphatidic acid (LPA) metabolism as controller of EMT process through the fine regulation of its synthesis and degradation enzymes. This pathway is upstream regulated by Autotaxin (ATX), an ectonucleotide pyrophosphatase/phosphodiesterase (ENPP) encoded by the *ENNP2* gene. The main ATX function is to promote the conversion of lysophosphatidilcoline (LPC) to LPA^40^ (Fig. 7).

Under physiological conditions, LPA repairs damaged tissues by stimulating cell growth, migration, survival, and angiogenesis^45^. Conversely, dysfunctional ATX and LPA signaling is found in several cancer, where ATX is among the top 40 most up-regulated genes^42^. In literature, only two studies have associated LPA to the induction of EMT in ovarian cancer cells^48,49^. In particular, it has been reported that LPA induced the transcription of EMT-related genes through the activation of HIF1α^49^. To the best of our knowledge, this is the first study to consider LPA metabolism as putative controller of EMT process. Indeed, the RNA-seq revealed that EMT (mAEC) promotes the upregulation of LPA synthesis whereas its inhibition (eAEC) induces the upregulation of LPA degradation enzymes. To this regard, AXT mRNA is among the main up-regulated transcripts (|fold change| = 1.74069) in cells that have experienced EMT (mAEC).

The homeostasis of LPA is further regulated by lipid phosphate phosphatases (LPPs), a class of enzymes encoded by *PLPP* genes that catalyze LPA hydrolysis into monoacylglycerol (MAG)^41^ (Fig. 7). Here all the five PLPP mRNAs isoforms (PLPP1–5) are identified as local hubs of eAEC network, thus suggesting a specific role in AXT/LPA signaling pathway modulation and in regulating EMT inhibition. In accordance with the present results, LPPs were found selectively down-regulated in several lung cancer patients where AXT/LPA pathways was aberrantly up-regulated^41^. In summary, the balance between LPA production (ATX-mediated) and degradation (LPP-induced) seems to have a key role in defining epithelial or mesenchymal fate of AEC.

The interaction of LPA with its receptors (LPAR) promotes the activation of phospholipase D1/2 (PLD1/2) which, in turn, produces phosphatidic acid (PA) from phosphatidylcholine (PC)^40^ (Fig. 7). PA represents a lipid mediator able to activate a variety of signaling^40,50^. PA degradation is also regulated by enzymes called lipins, encoded by *LPIN* genes^38,40^. Similar to PLPP genes, the present global genome analyses converge to identify the three isoforms of LPIN mRNA as local hubs of eAEC networks, even though no substantial mRNA up-regulation was recorded. Finally, intracellular LPA (iLPA) can be alternatively produced by cytosolic phospholipase A2 (cPLA2), using PA as substrate^38^ (Fig. 7). As the extracellular counterpart, iLPA hydrolysis is however under the control of LPPs^38^ (Fig. 7). In conclusion, all these findings lead to the hypothesis that LPA degradative pathways represents a key step driving the EMT inhibition in AEC.

Recently, ATX/LPA pathway activation was demonstrated to increase the stabilization and the nuclear translocation of Nrf2^42^ (Fig. 7). The function of Nrf2 is alternatively inhibited through its binding with Kelch-like ECH-associated protein 1 (Keap1), a member of Kelch-like (KHLH) gene family, also known as KLHL19^43^. In this study, only eAEC possess a significant high mRNA expression of KLHL14, another member of KLHL gene family. Little is known about the specific role of this gene, but its molecular structure presents many similarities with Keap1^43,44^. In physiological condition, Keap1 (or KLHL19) binds actin filaments and sequestrates Nrf2 into the cytoplasm. After oxidative stimuli, Keap1 conformational changes led to the nuclear translocation of Nrf2, wherein it induces the transcription of phase II detoxifying enzymes, such as GST and NQO1^51^ (Fig. 7). Here, it was demonstrated that exclusively mAEC show significant high GSTA1-1 and GSTM3 mRNA levels. Moreover, both genes are local hubs of mAEC network while GSTM3 is also a bottleneck, thus suggesting a driving role in the induction of EMT. In agreements with these results, GSTA1-1 and GSTM3 have been recognized as stimulatory respect proliferation, apoptosis evasion, metastatic process and EMT-related genes induction^52–54^.

There is few information on KLHL14 as EMT driver gene. As KLHL member, KLHL14 recognized an actin binding domain and a BTB domain which could be associated to a variety of cellular mechanisms such as control of cytoskeletal organization, ion channel gating^55^, transcription suppression and protein targeting for ubiquitination^43^. By translating these evidences to KLHL14, a putative mechanism could be hypothesized in which this protein regulates the activation of Nrf2 in a Keap1-like fashion (Fig. 7). Even if the present results point on KLHL14, further studies are needed in order to assess its role and of LPA/Nfr2 axis in EMT.

KNCE3 is another putative controller gene driving EMT inhibition in AEC model. The KCNE gene family encodes for five potassium (K^+^) channel regulatory β subunits^56^ that are found associated with KCNQ1 Kv α subunit^56^. Depending on the KCNE subunits, KCNQ1 gate opening is differently modulated. Interestingly, KCNE3, unlike other β subunits, locks KCNQ1 channel opened by removing almost completely its voltage dependent activation^56^. This results into a constitutive activation of KCNQ1 K^+^ channel^57^. Recent evidences demonstrated a steroid regulation of KCNQ1-KCNE3 channel. In particular estrogens induce KCNE3 downregulation, thus increasing the binding of other β subunits^57,58^. Conversely, Santos J. S. and colleagues found that progestin R5020 is able to induce high levels of KCNE3 mRNA in epithelial organoids derived from mammary gland^59^. Similarly, in the present study exclusively P_4_-treated eAEC show high KCNE3 mRNA expression. Even if the biological function of this gene has to be further clarified, several evidences indicate an involvement of similar potassium channels (e.g. KNCJ2, also known as Kir2.1) in the processes of cytoskeletal remodeling^45^. Therefore, future experiments are needed to demonstrate whether the upregulation of KCNE3 (with consequent constitutive channel opening) could eventually be involved in the cytoskeletal remodeling observed during EMT. Finally, a synergic action between KCNQ1-KCNE3 and Nrf2 should not be excluded. Indeed, nuclear translocation Nrf2 is also associated to an increase of KCNB1 β subunit mRNA transcription, thus suggesting that Nrf2 transcriptionally regulates K^+^ channel^46^. This discovery could open a new horizon in the comprehension of such a complex biological process linking the extracellular and intracellular signaling pathways of EMT.

In conclusion, in this study the transcriptomic changes occurring during the EMT in AEC were comprehensively profiled. Novel putative EMT regulators may represent a new branch point for the understanding of this crucial process with the potential to drive the development of new treatment strategies. Once understand the biological role of these modulating EMT genes, they may offer new potential therapeutic targets for cancer, especially in the context of that malignant transformations that are refractory to the conventional treatments.

## Materials and Methods

### Sample collection, cell culture and phenotypical characterization

The detailed material and procedures used for AEC isolation and characterization were performed according to Canciello *et al*.^26^, and Canciello *et al*.^47^. For detailed protocol of cell isolation, culture and reagents see Supplementary File S6.

### RNA extraction, quantification and qPCR validation

For each sample, 1 µg of total RNA was extracted using RNeasy Mini kit (Quiagen) as reported elsewhere^54^. To evaluate the RNA concentration, all samples were measured using a fluorescence-based RNA quantification approach (Qubit^TM^ RNA HS Assay, Life Technologies, Thermo Fisher Scientific Inc.).

### Sample library preparation and sequencing

The sequencing libraries were built using a standard Illumina RNA-seq protocol (https://www.illumina.com). Briefly, the mRNA was collected using poly-T oligo attached magnetic beads. After purification step, the mRNA was fragmented and was copied-back into first strand cDNA using SuperScript II (Invitrogen) and random primers (Illumina). Next, the second strand cDNA was synthesized by adding buffer dNTPs, DNA Polymerase I and RNase H. Following adenylation of 3′ ends of cDNA fragments, Illumina paired-end adapters were ligated to prepare for hybridation. The mRNA libraries were loaded onto NextSeq. 500/550 High Output Cartridge v2, 150 cycles kit (Illumina) and run on Illumina NextSeq. 500 platform to generate 75 bp paired-end reads.

### Sequencing data processing

To assess the quality of the obtained sequences, the raw reads, were subjected to quality check and trimmed using fastQC (https://www.bioinformatics.babraham.ac.uk/projects/fastqc) and Trimmomatic^60^ tools, respectively. Clean reads were aligned to the Ensembl ovine reference genome (Oar_v3.1.89) using Bowtie2^61^ and TopHat2^62^ software, imposing not more than two mismatches. Unmapped reads were excluded from downstream analyses.

### Differentially expressed genes (DEGs) analysis

The mapped reads were used as input for Cufflinks/Cuffdiff^63^ pipeline to quantify the gene expression levels between the two cell populations. In particular, it was imposed that only those genes with an adjusted p-value (q-value) lower than 0.05 and with a |log2_ratio| ≥ 1 showed a statistically significant expression difference and, therefore, were classified as differentially expressed among the two cell populations. The gene expression level was normalized using fragments per kilobase per million reads (FPKM) method, then allowing direct comparison among samples. The DEGs distribution and quality plots were produced by the DESeq. 2 package^64^ within R (www.R-project.org).

### GO and KEGG enrichment

The DEGs were then annotated to the Gene Ontology (GO; https://www.geneontology.org) and the Kyoto Encyclopedia of Genes and Genomes (KEGG; https://www.genome.jp/kegg/) databases. The GO enrichment analysis was used to identify the main functions of the DEGs according to their GO terms. DEGs were independently annotated in the three main GO ontologies (Biological process, Cellular component and Molecular function) and successively it was carried out the enrichment analysis using the Fisher’s test, implemented in the topGO R-package^65^. Only those GO terms having an adjusted p-value (q-value) ≤ 0.01 were considered to be significantly enriched. Similarly to the previous approach, the KEGG pathways enrichment analysis was carried out using a hypergeometric test implemented in the edgeR package^66^. The pathways with a q-value ≤ 0.05 were recognized to be significantly enriched.

### Gene-gene interaction network analysis

Based on the enriched KEGG pathways obtained from the previous analysis, it was built a gene-gene interaction network for each population. In particular, the KEGG graph R-package was used^67^ to convert the mAEC and eAEC enriched pathways into graphs where nodes are molecules and edges represent the relationship between them. To reduce complexity, the chemical compounds were removed from the final graphs. The resulting networks were displayed using the Cytoscape platform 3.6.0 version (http://www.cytoscape.org) and main connected component (MC) extracted. To assess the networks’ topology, the topological parameters were automatically measured using the Network Analyzer plug in, considering the networks as directed. According to Bernabò *et al*.^68^, the *hubs* within MC_mAEC and MC_eAEC were identified as the nodes with a degree at least one standard deviation above the network mean. Next, attention was focused on nodes with the highest relative betweenness centrality identifying the top 200 *bottleneck* genes using the Cytoscape CytoHubba plug in.

### Kernel density estimation

The KDE analysis was performed using Past3 software, according to Bernabò *et al*.^68^ and Ordinelli *et al*.^36^.

## Supplementary information

Supplementary File S1.

Supplementary S6.

Dataset S2.

Dataset S3.

Dataset S4.

Dataset S5_A.

Dataset S5_B.

## Data Availability

The datasets generated during and/or analysed during the current study are available from the corresponding authors on reasonable request.
