# Supplementary material for: Transcriptomic and computational analysis identified LPA metabolism, KLHL14 and KCNE3 as novel regulators of Epithelial-Mesenchymal Transition
Source: Sci Rep. 2020 Mar 6;10:4180. doi: 10.1038/s41598-020-61017-y (PMC7060278; doi:10.1038/s41598-020-61017-y)
Supplement: Supplementary file 1 — Supplementary File S1. [file 41598_2020_61017_MOESM1_ESM.docx]

**Transcriptomic and computational analysis identified LPA metabolism, KLHL14 and KCNE3 as novel regulators of Epithelial-Mesenchymal Transition**

Di Lollo V.^*^, Canciello A.^*^ (equally contributing authors), Orsini M., Bernabò N., Ancora M., Di Federico M., Curini V., Mattioli M., Russo V., Mauro A., Cammà C., Barboni B.

**Supplementary File S1. Filtering procedure of RNAseq data.**

The filtering procedure aimed at guaranteeing a high-quality dataset in terms of annotation and reliable expression values. It is schematically shown in Figure2-panel B.

After quality control and trimming, the TopHat2/Cufflinks pipeline was applied to analyze transcriptome of mAEC and eAEC, returning expression levels for **33,150 genomic *loci***.

First, the loci having a stable identifier in the *Ovis aries* Ensembl database ([https://www.ensembl.org](https://www.ensembl.org/)) were retained, excluding those not having specific genomic feature in the database.

The resulting **26,166 *loci*** were grouped based on their correspondent gene type with the aim of focusing on protein-coding genes only. This second step enabled to exclude from downstream analysis, ribosomal genes (320 *loci*), pseudogenes (378 *loci)* and generally non-coding genes such as miRNA, snoRNA, snRNA etc… (5,280 *loci*).

Of the resulting **23,517 protein-coding *loci***, only those showing a unique Ensembl ID (**20,297**) were retained.

This subset, showing a consistent annotation, was then filtered by considering the levels of expression. The *loci* for which the TopHat2/Cufflinks pipeline returned FPKM equal to 0 in both mAEC and eAEC were discarded, then retaining a dataset consisting of **16,847 protein-coding *loci***.

By comparing the transcriptional profiles of the two populations, different patters emerged.  Indeed, as shown in the Figure2-panel C, even though most of the genes resulted being commonly expressed among the two populations (15,708), several genes exclusively expressed in mAEC (481 genes) or in eAEC (658 genes) were found.

To focus on genes characterizing each cell population, exclusively those with a q-value lower than 0.05 and exhibiting a fold change higher than |log2fold| ≥ 1 were henceforward considered.

This established a genes subset that showed statistically significant differential expression between mAEC and eAEC (Figure2-panel C), rendering a total of 1,259 differentially expressed genes (DEGs).

In particular, the expression levels of 495 DEGs resulted being increased in the mAEC respect to the eAEC whereas, on the other hand, 753 DEGs were over-expressed in the eAEC respect to mAEC.

Top 30 DEGs, characterizing the mAEC and eAEC transcriptome landscape, were listed in TableS1 panel A-B ranked by fold change values. In particular, the attention has been focused on those genes expressed exclusively in one of the two populations, identifying 5 genes over-expressed in mAEC only (blue boxes in TableS1 panel A**)** and 9 genes over-expressed in the eAEC only (red boxes in TableS1 panel B).

| **Gene ID**  A | **Gene description** | **mAECvseAEC** | **Q-value** | B | **Gene ID** | **Gene description** | **eAECvsmAEC** | **Q-value** |
| --- | --- | --- | --- | --- | --- | --- | --- | --- |
| **SLN** | **sarcolipin** | **∞** | **0.00312453** |  | **KLHL14** | **kelch like family**  **member 14** | **∞** | **0.000137** |
| **TFF3** | **trefoil factor 3** | **∞** | **0.00312453** |  | **ENSOARG00000003845** | **-** | **∞** | **0.000137** |
| **SMPX** | **small muscle**  **protein X-linked** | **∞** | **0.00312453** |  | **KCNE3** | **potassium voltage-gated channel**  **subfamily E regulatory subunit 3** | **∞** | **0.000137** |
| **ENSOARG00000000376** | **-** | **∞** | **0.0414459** |  | **ENSOARG00000020985** | **natural killer cells**  **antigen CD94-like** | **∞** | **0.000137** |
| **C5orf58** | **chromosome 5 open**  **reading frame 58** | **∞** | **0.0414459** |  | **COL9A1** | **collagen type**  **IX alpha 1 chain** | **∞** | **0.000137** |
| PPP6R1 | protein phosphatase 6  regulatory subunit 1 | 5.98565 | 0.00013681 |  | **ENSOARG00000019504** | **-** | **∞** | **0.000137** |
| ENSOARG00000000342 | - | 5.72703 | 0.00013681 |  | **ENSOARG00000010151** | **-** | **∞** | **0.003335** |
| ENSOARG00000010685 | - | 5.63158 | 0.00406312 |  | **ENSOARG00000005173** | **-** | **∞** | **0.003335** |
| ENSOARG00000004823 | Dermokine | 4.5236 | 0.0246216 |  | **ENSOARG00000014113** | **-** | **∞** | **0.043163** |
| CD53 | CD53 molecule | 4.30832 | 0.00013681 |  | **ENSOARG00000005970** | - | 6.03602 | 0.000137 |
| SPINK5 | serine protease inhibitor  Kazal-type 5 | 4.2724 | 0.00013681 |  | FBN3 | fibrillin 3 | 5.80043 | 0.000137 |
| KRT4 | keratin 4 | 4.23368 | 0.00013681 |  | PLEKHB1 | pleckstrin homology  domain containing B1 | 5.76114 | 0.000137 |
| ENSOARG00000005384 | - | 4.04497 | 0.00013681 |  | WDR72 | WD repeat  domain 72 | 5.4564 | 0.000137 |
| KRT78 | keratin 78 | 4.01422 | 0.00013681 |  | ATP6V1C2 | ATPase H+ transporting  V1 subunit C2 | 5.36576 | 0.000137 |
| S100A8 | S100 calcium  binding protein A8 | 3.94297 | 0.00013681 |  | SLCO4C1 | solute carrier organic anion  transporter family member 4C1 | 5.28207 | 0.005715 |
| SNCAIP | synuclein alpha  interacting protein | 3.93601 | 0.00013681 |  | ARFGEF3 | ARFGEF family member 3 | 5.27904 | 0.001351 |
| ENSOARG00000019719 | ribonuclease A  family member 2 | 3.85311 | 0.00013681 |  | HPN | hepsin | 4.79451 | 0.000517 |
| S100A9 | S100 calcium  binding protein A9 | 3.71562 | 0.00013681 |  | GCNT1 | glucosaminyl (N-acetyl)  transferase 1, core 2 | 4.63986 | 0.000137 |
| MUC15 | mucin 15,  cell surface associated | 3.65225 | 0.0046902 |  | SLC6A12 | solute carrier family 6  member 12 | 4.59679 | 0.000517 |
| ENSOARG00000014766 | - | 3.64296 | 0.00013681 |  | STMN4 | stathmin 4 | 4.56442 | 0.000137 |
| KRT13 | keratin 13 | 3.63142 | 0.00013681 |  | GPA33 | glycoprotein A33 | 4.53705 | 0.000137 |
| TMPRSS11D | transmembrane  serine protease 11D | 3.58246 | 0.00013681 |  | LIPH | lipase H | 4.51032 | 0.000137 |
| S100A12 | protein S100-A12 | 3.57572 | 0.00013681 |  | SLC37A2 | solute carrier family 37  member 2 | 4.48551 | 0.000137 |
| DPP4 | dipeptidyl peptidase 4 | 3.53863 | 0.00013681 |  | ENSOARG00000005914 | - | 4.40777 | 0.000137 |
| SRGN | Serglycin | 3.48207 | 0.00013681 |  | SOWAHB | sosondowah ankyrin repeat  domain family member B | 4.37687 | 0.000137 |
| BLNK | B cell linker | 3.44667 | 0.00013681 |  | ENSOARG00000008811 | - | 4.35546 | 0.000137 |
| FABP4 | fatty acid binding protein 4,  adipocyte | 3.43693 | 0.00099958 |  | ADAMTS12 | ADAM metallopeptidase with  thrombospondin type 1 motif 12 | 4.31401 | 0.000137 |
| TMPRSS11B | transmembrane protease  serine 11B-like protein | 3.4245 | 0.00013681 |  | SEMA6A | semaphorin 6A | 4.28251 | 0.000137 |
| ENSOARG00000009963 | serum amyloid A  protein-like | 3.32484 | 0.00013681 |  | FGF13 | fibroblast growth factor 13 | 4.25009 | 0.000137 |
| PNPLA1 | patatin like phospholipase  domain containing 1 | 3.29989 | 0.00013681 |  | ATP10B | ATPase phospholipid  transporting 10B (putative) | 4.24825 | 0.000137 |

**Table S1.** DEGs characterizing the mAEC and eAEC transcriptome landscape. The table shows the top 30 over-expressed genes in mAEC (A) and in eAEC (B). For each list of genes, the gene name or the unique code provided by ENSEMBL database (Gene ID), the characteristic gene description, the log-ratio of gene's expression values between the two populations (log2(fold change)) and the corrected p-value (Q-value) are displayed. The ∞ symbol indicates that the genes are over-expressed exclusively in the relative population. To detect DEGs, q-value ≤ 0.05 and |log2(foldchange)| ≥ 1 were set as threshold.
