# Supplementary material for: Transcriptomic and computational analysis identified LPA metabolism, KLHL14 and KCNE3 as novel regulators of Epithelial-Mesenchymal Transition
Source: Sci Rep. 2020 Mar 6;10:4180. doi: 10.1038/s41598-020-61017-y (PMC7060278; doi:10.1038/s41598-020-61017-y)
Supplement: Supplementary file 2 — Supplementary S6. [file 41598_2020_61017_MOESM2_ESM.docx]

**Transcriptomic and computational analysis identified LPA metabolism, KLHL14 and KCNE3 as novel regulators of Epithelial-Mesenchymal Transition**

Di Lollo V.^*^, Canciello A.^*^ (equally contributing authors), Orsini M., Bernabò N., Ancora M., Di Federico M., Curini V., Mattioli M., Russo V., Mauro A., Cammà C., Barboni B.

**Supplementary File S6. Cell culture methods.**

1. **Isolation and cell culture method of AEC**
   1. **Materials and Reagents**

Antibiotics buffer for membrane isolation (use outside the laminal flow hood): 1% of 10000 UI/mL Penicillin-Streptomycin in Sodium chloride solution suitable for cell culture. Store at room temperature.

Antibiotics buffer for cell isolation (use inside the laminal flow hood): 1% of 10000 UI/mL Penicillin-Streptomycin in Phosphate Buffered Saline (PBS) without calcium and magnesium, suitable for cell culture. Store at room temperature.

P_4_ stock solution: Weight 100 mg of P_4_ and transfer in a sterile 15 mL tube. Add absolute ethanol to a volume of 10 mL and gently mix the solution. Work under fume hood. The concentration of P_4_ stock solution is 31.8 mM. Store at 4 °C.

P_4_ working solution: Dilute 5 mL of P_4_ stock solution in 5 mL of absolute ethanol and gently mix the solution. Work under fume hood. The concentration of P_4_ working solution is 15.9 mM. Store at 4 °C.

AEC Growth Medium: Alpha minimum essential eagle medium supplemented with 20% Fetal Bovine Serum (FBS), 1% Ultraglutamine, 10000 UI/mL Penicillin-Streptomycin, and 2.5 μg/mL Amphotericin. Gently mix the solution. Filter the medium in a 0.22-μm filter and equilibrate in an incubator at 38.5 °C, 30 min before the use.

AEC Growth Medium supplemented with Progesterone (P_4_): Alpha minimum essential eagle medium supplemented with 20% Fetal Bovine Serum (FBS), 1% Ultraglutamine, 10000 UI/mL Penicillin-Streptomycin, and 2.5 μg/mL Amphotericin. Add P_4_ at final concentration of 25 μM by discarding the equivalent volume of Growth Medium to exactly maintain the correct P_4_ concentration. Gently mix the solution. Filter the medium in a 0.22-μm filter and equilibrate in an incubator at 38.5 °C, 30 min before the use.

- 1. **Isolation of AEC from amniotic membrane**

**1.** Carefully prepare the uterus and the incision site with denatured alcohol.

**2.** Open the uterus wall with the aid of surgical forceps. Afterwards, gently separate placenta from the uterus by manually detaching the cotyledons from caruncles.

**3.** Once the placenta is isolated from the rest of the uterus, roughly peel off with surgical and watchmaker tweezers the chorioallantois from the amnion.

**4.** Cut amnion pieces with the aid of surgical tweezers and forceps and put them into Antibiotics buffer for membrane isolation.

**5.** Move the amnion pieces under laminal flow hood and put them into Antibiotics buffer for cell isolation.

**6.** Working into a 10 cm petri dish filled with Antibiotics buffer for cell isolation, divide the amnion in smaller pieces of about 3–5 cm of length by using sterile watchmaker tweezer and scalpel.

**7.** Manipulate under a stereomicroscope in order to finely remove the residual parts of chorioallantois from the amnion with the aid of fine watchmaker forceps.

**8.** Dissect amnion with sterile watchmaker tweezer and scalpel to get tissue pieces of about 1 cm^2^ or less.

**9.** Rinse three times for 15 min the amnion pieces with Antibiotics buffer for cell isolation.

**10.** Incubate amnion pieces into trypsinization flasks. Add 0.25% Trypsin-EDTA solution and a magnetic stir bar. Place the trypsinization flasks on a magnetic stirrer in 38 °C water bath for 30 min, with consistent agitation.

**11.** Add FBS 10% (v/v) to cell suspension in order to inactivate trypsin. Collect cell suspension, filter through a 40-μm cell filter and pour into a 50 mL tube. Centrifuge the cell suspension at 300 x *g* for 10 min.

**12.** Discard the supernatant and resuspend the pellet in pre-warmed and equilibrated Growth Medium.

**13.** Dilute 1:2 cell suspension in Trypan Blue into a 0.5 micro tube and gently pipette the suspension. Count the cell suspension by using Bürker counting chamber.

**14.** Seed the cells at the final concentration of 3 × 10^3^ cells/cm^2^ in Growth Medium supplemented with 25 μM of P_4_ in order to preserve the epithelial phenotype (epithelial AEC: eAEC). On the other hands, AEC cultured in Growth Medium without P_4_ experience epithelial to mesenchymal transition within three cultural passages (mesenchymal AEC: mAEC).

**15.** Use 100 mm dishes (Thermo Fisher Scientific, 150350) for the first passage and then use 175 cm^2^ flask (Thermo Fisher Scientific, 159910) for further amplifications. Do not treat culture vessels with other cell substrates.

**16.** Carefully mix the dish by gentle agitation to obtain an equal seeding of the cells.

**17.** Incubate the culture dish in incubator at 38.5 °C in 5% CO2.

**18.** Refresh AEC Grow Medium every two days.

**19.** Dissociate cells with 0.25% Trypsin-EDTA when they reach 75-80% of confluency.
